# The Qualitative Descriptive Approach in International Comparative Studies: Using Online Qualitative Surveys

**DOI:** 10.15171/ijhpm.2017.142

**Published:** 2017-12-23

**Authors:** Brayan V. Seixas, Neale Smith, Craig Mitton

**Affiliations:** ^1^School of Population and Public Health, Faculty of Medicine, University of British Columbia, Vancouver, BC, Canada.; ^2^Centre for Clinical Epidemiology & Evaluation, Vancouver Coastal Health Research Institute, University of British Columbia, Vancouver, BC, Canada.

**Keywords:** Qualitative Description, Online Qualitative Survey, International Comparative Studies, Health Policy Analysis

## Abstract

International comparative studies constitute a highly valuable contribution to public policy research. Analysing different policy designs offers not only a mean of knowing the phenomenon itself but also gives us insightful clues on how to improve existing practices. Although much of the work carried out in this realm relies on quantitative appraisal of the data contained in international databases or collected from institutional websites, countless topics may simply not be studied using this type of methodological design due to, for instance, the lack of reliable databases, sparse or diffuse sources of information, etc. Here then we discuss the use of the qualitative descriptive approach as a methodological tool to obtain data on how policies are structured. We propose the use of online qualitative surveys with key stakeholders from each relevant national context in order to retrieve the fundamental pieces of information on how a certain public policy is addressed there. Starting from Sandelowski’s seminal paper on qualitative descriptive studies, we conduct a theoretical reflection on the current methodological proposition. We argue that a researcher engaged in this endeavour acts like a composite-sketch artist collecting pieces of information from witnesses in order to draw a valid depiction of reality. Furthermore, we discuss the most relevant aspects involving sampling, data collection and data analysis in this context. Overall, this methodological design has a great potential for allowing researchers to expand the international analysis of public policies to topics hitherto little appraised from this perspective.

## Introduction

International comparative studies may contribute enormously to public policy research. Understanding the distinct manners in which a certain issue may be tackled provides better comprehension of the problem itself as well as useful insights on the design of institutional responses. For instance, the works of Hall and Lamont,^1^ Stuckler and Basu,^2^ and Schrecker and Bambra^3^ consistently show through cross-national comparisons how certain economic policies have profoundly impacted on population health.

However international comparisons pose demanding data collection challenges. Much of the work performed in such studies relies on existing databases kept by international agencies, third sector organizations or research institutions (eg, World Bank, OECD, WHO, UN). For studies on some topics, however, there is no reliable database from which the necessary information might be retrieved. Thus, it is vital that alternative, methodologically rigorous approaches emerge.

Here we discuss the use of online qualitative surveys as a tool to overcome the difficulties of conducting comparative studies on public policies in different national jurisdictions. The basic idea is that instead of relying on institutional websites, publicly available policy documents or well-established databases to understand how certain policies have been addressed, we could question key stakeholders (such as policy-makers, public servants or researchers involved with the topic in question) from each relevant national context in order to retrieve essential information directly from them.

Given that the diversity of responses can be totally unanticipated by researchers, it is necessary to have a tool that is open enough to allow any type of information to be captured, for which purpose qualitative methodologies are highly appropriate. However, taking into account that the main objective of this type of research is to obtain comparable information from a potentially large number of different countries, the methodology needs also to pre-structure responses to a sufficient extent to allow for viable and efficient data reduction and analysis. And for this particularity, a qualitative description approach through the use of online qualitative surveys, ie, structured questionnaires with open-ended questions, seems to be an interesting solution.

Thus, this paper aims to provide a discussion of the theoretical reasoning underlying the qualitative description approach – presenting as an adequate solution to the tensions noted above – as well as practical insights on the development of such work within the realm of international comparative studies on public policies.

## Theoretical Reasoning

According to Sandelowski,^4^ *basic* or *fundamental* qualitative description differs from other types of qualitative research, such as grounded theory, ethnography, phenomenology or narrative analysis, in the sense that it is — as the label suggests — essentially descriptive rather than interpretive in focus. This does not mean that a qualitative descriptive approach lacks interpretive efforts or that it intends a supposedly neutral depiction of reality. Qualitative description represents the methodological category that has the least level of inference among the qualitative methods, one that allows “*the reading of lines, as opposed to reading into, between, over or beyond the lines.*”^5^ However, it should not be understood as a low-quality approach or solely as an entry-point to really deep research. “*There is nothing trivial or easy about getting the facts, and the meanings participants give to those facts, right and then conveying them in a coherent and useful manner.*”^4^ Such qualitative description must be viewed as a valuable end-product in itself, and not simply as an entry-point.

We propose the use of on-line surveys as a way of operationalizing qualitative description in international comparative studies, allowing the retrieval of information on governmental/institutional efforts to develop, implement and evaluate public policies based on the reports of involved stakeholders who draw upon their own situated experience and knowledge. Within this context, we offer a reflection emerging from Kvale’s metaphor^6^ on the role of a researcher. Kvale presents two ideal types: the researcher as a miner and the researcher as a traveller. For the former, the reality is out there waiting to be discovered. The job of a miner is then to find the precious stones, the gems, ie, the pieces of reality that have value in a given social setting. Thus, the miner-researcher operates under a predominantly positivist framework. On the contrary, the traveller is experiencing the reality herself. There is no separation of what a traveller has to tell us about the reality from the actual reality. Therefore, the traveller-researcher is not a collector of pieces of information, but rather she/he is the proper constructor of the pieces. This type of researcher marches mainly under a socio-constructivist paradigm.

Kvale’s metaphor is indeed incredibly insightful to reflect on the role of the qualitative researcher in general, which should not be understood as either miner or traveller, but as an enterprise with a predominance of one or the other role. Yet for qualitative descriptive studies particularly, we suggest that another metaphorical representation can be even more powerful. Here we propose that the role of a researcher involved in qualitative descriptive efforts is that of a *composite sketch artist*. The underlying idea is that this artist has the role of depicting a ‘reality’ based on the reports of the witnesses. In other words, the artist has the duty of drawing a picture that is in accordance to the memories of the witnesses, rather than substituting his/her own speculation in its stead. The artist inevitably has her/his own images in mind, but the aim is to capture the understanding of the other—a picture that the witnesses would agree represents the reality they experienced. Contextualizing this for the field of policy research, the role of the researcher conducting qualitative descriptive study is to retrieve information from stakeholders about their own experiences with the institutions in order to reconstruct the actual governmental designs of public policies or organizational management systems. Thus, the method employed has to faithfully draw the picture upon which most of the interviewees from a given setting will agree.

This metaphor leads us to reflect on the concepts of descriptive and interpretive validity, as elaborated by Maxwell.^7^ For Maxwell, descriptive validity refers to the accurate, ‘correct’ or faithful use of the factual aspects of data. It is predominantly related to the elements “pertaining to physical and behavioral events that are, in principle, observable.”^7^ For example, policy content and the means by which policy is enacted within given political jurisdictions. Thus, the large majority of the work conducted in qualitative descriptions are almost exclusively circumscribed to this level of interpretation and validity. In other words, descriptive validity deals with how the composite-sketch artist treats the information provided by the witness. It does not mean that the researcher would actually work as a copier or a mere reproducer – impossible as he/she is not within the head of the observer and does not share the experience in question. The important thing to note here is that qualitative description is not an inference-free approach, but rather the methodological work of least inference among categories of qualitative work. In the context of international comparative studies, the researcher has to ‘keep close to the surface’ of the information provided by the stakeholders in order to appropriately describe the local systems of management or public policies.

Albeit the fundamental concern of qualitative descriptive studies is to provide a sort of report of events, institutional structures, and commonly observable behaviors, it is also important that researchers account for the meaning of these things for the people studied. It does not signify that qualitative description will dive deeply into the web of meanings in which subjects are constantly moving, but there has to be at least a conscious movement of acknowledging this phenomenon in order to obtain a valid drawing of the reality. This is what Maxwell calls interpretive validity.^7^ Thinking in terms of our proposed metaphor, the composite-sketch artist needs to take into consideration what pieces of information provided by witnesses actually can mean to them. So, the witness may say that the crime perpetrator has big green eyes, but although this constitutes factual information, this is not enough in order to draw the actual eyes that would be recognized by the witness. The understood meaning of ‘big’ emerges on paper through the efforts of the sketch artist. It is necessary to take account some level of interpretive data, though just as long as it indeed helps the ‘reconstruction of reality.’

The next section will focus on the more practical details of developing an online survey as a qualitative descriptive endeavour for international comparative studies.

## Methodological Issues

As Sandelowski^5^ points out, qualitative description is a *distributed residual category* and, as such, it makes visible the “*porous lines between qualitative and quantitative description (…) and between the erosion and re-invention of method*” (p. 82). In other words, this category of inquiry may incorporate elements from quantitative and qualitative methodologies and, thus, serve as an innovative research tool. In the particular case of obtaining information from different national contexts, a qualitative description approach allows collection of data that will be analysed not only from the perspective of traditionally qualitative methodologies, but also from a more quantitative lens, making possible a quasi-statistical analysis of content, providing an overall summary of the findings.

## Sampling

For this type of research, we propose a combination of purposeful sampling strategies – here we rely on the classification system developed by Patton.^8^ At the initial level of sampling, ie, the country-level, it is important to ensure comparability among the selected nations. This is extremely important for the validity of the quantitizing stage of data analysis, which should report a numerical summary of the data and observed patterns. For this level of sampling, hence, it is important to combine two strategies: homogeneity sampling and criterion sampling. For instance, we could select countries by the number of inhabitants or we could decide to include countries only above or below a given value of gross domestic product (GDP) per capita.

Subsequently, sampling may focus on using strategies to guarantee that there is meaningful variation within the sample and that politically important cases are not missing. For example, it may be appropriate to include cases with distinct institutional models, such as countries with parliamentary and presidential systems, or countries with centralized and decentralized responsibilities for a given public service.

Once the countries to be included in an international comparative study are determined, researchers need to identify individual informants. While an explicit sampling frame (eg, a directory of government department heads) may sometimes be available, strategies such as snowball sampling and convenience sampling may be required in order to make the study viable. Figure depicts this whole process of sampling within the context of using a survey as a qualitative descriptive tool to study public policies across countries.

**Figure:**
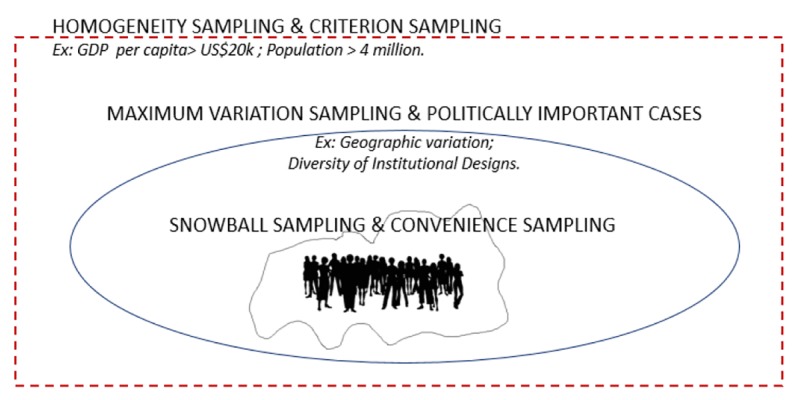
Purposeful Sampling Strategies for Qualitative Description in International Comparative Studies.

### Data Collection

As aforementioned, the main objective of data collection within this context is to obtain information about the institutional design of public policies. For this, researchers will rely on the reports of participants to reconstruct the ‘reality’ of each national scenario.

It is precisely at this point that the survey is the basic tool for collecting data. Respondents located in each national context would be invited to participate in an online qualitative survey. Researchers will have to circulate a structured survey instrument that allows participants to express their ideas on their own terms, but, at the same time, within a format that facilitates or guides the process of data analysis. Considering that qualitative description aims to record the fact, or in other words, to describe the things upon which most people would readily agree, the research team needs to develop an effective survey to engage participants in the description of the essential policy elements, without narrowing their possibilities of responses.

### Data Analysis

For Sandelowski,^4^ the analytic strategy of choice in qualitative description is *Qualitative Content Analysis*. This is a dynamic analytical tool intended to depict the informational content of the data.^9^ Although similar to quantitative content analysis, this is different because the codes are commonly generated from the data (ie, derived inductively) in the course of the study. In addition, in qualitative content analysis, the quantitizing phase (the stage when the coded data elements can be numerically organized) allows the researcher to go beyond the mere summarization of the manifest data (the information readily retrievable from the raw dataset). Inferring associations, depicting tendencies and making predictions provide insight into the latent content of data obtained (ie, the type of information that requires a deeper analytic effort to be revealed).

In the context of international comparative studies, other data analysis strategies may also prove fruitful. For instance, the gaps between qualitative and quantitative analyses can be bridged using Ragin’s Qualitative Comparative Analysis.^10^ This may be a valuable tool to investigate the generalizability of findings as well as the causal complexity of the variables encountered in the coded data.

## Discussion

By conducting qualitative descriptive studies with decision-makers, public servants and/or the local research community, it is possible for international comparative studies to use participants’ contextually-situated knowledge to depict the realities of different public policy contexts. The metaphor of the composite sketch artist can be a powerful device to guide methodological reflections, such as the notions of descriptive validity (the depiction of events as perceived by observers in their apparent sequence) and interpretive validity (the appropriate elicitation of the meanings attributed by the agents to those events). It illuminates the researcher’s role in presenting a picture of the topic investigated that most observers would likely agree with.

We argue that qualitative descriptive efforts are neither thick nor thin descriptions, in the sense used by Clifford Geertz.^11^ By his account, a thick description is an instrument that unveils the web of significance allowing the researcher to differentiate among ‘the conspiratorial winking,’ ‘the involuntary twitch,’ ‘the parodic-fake winking,’ and ‘the rehearsing winking,’ whereas the thin description is the mere report of people rapidly contracting the eyelids. A qualitative description is something else. A *fundamental* or *basic* qualitative descriptive endeavour would seek to describe, for instance, who are the ones supposedly winking, how many they are, how many times they wink, which other gestures are being done, where these people are situated, who else is present, etc. Basic or fundamental qualitative descriptive studies thus cannot be properly understood within this Geertzian dualistic epistemological framework. Its virtue as a qualitative category of inquiry that stand per se (despite its residual nature) is only acknowledged by inscribing it within other framings. A qualitative description could be understood as a comprehensive description, one that seeks to provide a detailed description of the findings more likely to generate consensus among observers.

In the voluminous literature on qualitative research methods, there is no comprehensive study with a systematic reflection about the use of online qualitative surveys in international comparative studies on public policies. Therefore, our current endeavour may provide a valuable contribution to the research community as a potential approach available to researchers for investigating comparative topics in a methodologically rigorous manner.

## Ethical issues

Not applicable.

## Competing interests

Authors declare that they have no competing interests.

## Authors’ contributions

BVS developed the paper’s central reasoning and crafted the first draft; NS provided extensive support in reviewing the text and offering fundamental contributions; CM supervised the whole work and provided meaningful contributions up to its final version.

## Authors’ affiliations

^1^School of Population and Public Health, Faculty of Medicine, University of British Columbia, Vancouver, BC, Canada. ^2^Centre for Clinical Epidemiology & Evaluation, Vancouver Coastal Health Research Institute, University of British Columbia, Vancouver, BC, Canada.
